# Optimization of Oil Sorbent Thermoplastic Elastomer Microfiber Production by Centrifugal Spinning

**DOI:** 10.3390/polym15163368

**Published:** 2023-08-11

**Authors:** József Kántor, Rudolf László Farmos, Attila Levente Gergely

**Affiliations:** Department of Mechanical Engineering, Faculty of Technical and Human Sciences, Sapientia Hungarian University of Transylvania, 540485 Târgu-Mureş, Romania; kantorjozsef@ms.sapientia.ro (J.K.); farmos_rudolf@ms.sapientia.ro (R.L.F.)

**Keywords:** fiber, SIBS, centrifugal spinning, oil sorption, thermoplastic elastomer

## Abstract

Fibrous structures are promising candidates for oil–water separation applications. In this study, we have produced poly(styrene-*b*-isobutylene-*b*-styrene) thermoplastic elastomeric fibers with the centrifugal spinning fiber production method. The optimal fiber production conditions were achieved when using a 25% *w*/*w* solution concentration in an 80/20 tetrahydrofuran/toluene (*w*/*w*) solvent system at 8000 rpm rotational speed. The produced fibers were bead-free and smooth-surfaced with a diameter of 3.68 µm. The produced fibers were highly hydrophobic and oleophilic, suggested by a water contact angle of 129° and the instantaneous absorption of the oil droplet. The oil absorption study showed fast absorption kinetics with 94% relative oil uptake after 1 min and a maximum of 16.5 g sunflower oil/g fiber. The results suggest that polyisobutylene-based thermoplastic elastomers could be promising alternatives in oil absorption applications.

## 1. Introduction

Large-scale oil spill events have a detrimental effect on the environment, which includes wildlife on the shorelines as well as marine ecosystems [1,2]. There are a multitude of methods that aim to contain the spills and collect the oil. These include mechanical methods (skimming, using sorbent materials), chemical methods (these usually complement the mechanical procedures by dispersing, gelling, de-emulsifying the oil), in situ burning, and bioremediation [3,4]. Sometimes, the cleanup method causes further damage in itself, especially if it involves the use of chemicals.

Sorbents are porous structures that can quickly absorb and retain large amounts of liquid. In oil collection applications, the most relevant properties of the materials in question are their hydrophobicity and oleophilicity. Natural fibers are usually cheap, but also have poor oil absorption performance. More efficient oil collection devices can be fabricated by creating open-cell foams and sponges, gels, fiber mats from synthetic polymers, and through the surface functionalization or coating of non-oleophilic materials [5].

Nano- and microfibrous mats have a high surface-to-volume ratio, and as such, they are an excellent choice for oil collection devices. A popular method to produce non-woven fiber mats is electrospinning. Electrospinning utilizes a strong electrostatic field (500–1000 V/cm) that stretches a polymer solution jet. The tensile force causes the thinning of the liquid jet, and at the same time, the solvent evaporates, leaving polymer fibers behind with diameters of 0.1–10 μm [6]. Most laboratory electrospinning setups utilize a single needle (producing a single polymer jet), and the typical volumetric flow rates used are in the 1–2.5 mL/h range [7,8]. Needleless electrospinning setups used for large-scale production can reach a fiber production rate of 40 g/h [9]. By the nature of the process, polymer solutions used with electrospinning have to be electrically conductive, thus limiting the type of polymers and solvents used. Oil sorption capacity as high as 900 g oil/g fiber has been reported for highly porous electrospun polystyrene (PS) fibers [10].

An emerging technology for the creation of micro- and nanofibers is centrifugal spinning. A small chamber containing the polymer solution is rotated at 4000–15,000 rpm. Nozzles are attached to the chamber in the radial direction, and during rotation, the polymer solution is expelled through these as a consequence of the arising centrifugal force [11]. Furthermore, the centrifugal force also acts as the tensile force responsible for elongating the polymer jet. Since there is no electrostatic field involved, the polymer solutions no longer have to be electrically conductive, as in the case of electrospinning. Some published data about centrifugally spun fibers show fiber diameters of 0.5–4 μm [12], 1.5–15 μm [13], and 0.6–1.5 μm [14], and volumetric low rates of 60–300 mL/h [12,13,14].

While electrospinning and centrifugal spinning are primarily used for fiber creation from polymer solutions, spinning from polymer melts can be performed as well [15,16]. There is also a separate fiber production method involving polymer melts called melt blowing, in which case a high-velocity air stream plays a key role in the fiber formation. It is most widely used with polymers that are difficult to dissolve (e.g., polypropylene), and the produced fibers can be in the 0.3–10 μm thickness range [17,18,19]. The most important advantage of melt spinning/blowing is that it does not require the use of organic solvents (preventing pollution or the energy expenditure for solvent capture), but in exchange, the heating of the polymer requires additional energy, and heat-sensitive compounds cannot be blended with the polymer.

There are a couple of studies already dealing with the creation of fiber mats by centrifugal spinning for oil collection applications. Doan et al. spun fibers with an average size of 4–6 μm from a 22% *w*/*w* PS solution in tetrahydrofuran (THF) and dimethylformamide (DMF) at 15,000 rpm and a flow rate of 100 mL/h. The produced fibers were porous, had a water contact angle (WCA) of 145°, and had an oil uptake of 30–50 g/g, depending on the porosity of the fibers [20]. Zhang et al. used centrifugal spinning to create porous PLA fibers from 6 to 8% *w*/*w* solution in chloroform. The procedure was performed at 4000 rpm (after an initial ramp-up in speed over 10 s), and resulted in the formation of 6 μm thick fibers. When the rotational speed was increased to 5000 rpm, the average fiber size decreased to 4 μm. The measured WCA was 116°, oil droplets were absorbed in 2 s, and the oil collection capacity was 25–30 g/g [21].

In the pursuit of finding suitable materials for oil collection applications, polymers with molecular structures similar to those of the oils come to mind. For aromatics, there is PS, for example, which is a popular choice for fiber production. When it comes to aliphatics polyethylene (PE), polypropylene (PP) and polyisobutylene (PIB) would be the potential choices. PE and PP are semi-crystalline and thus are difficult to make a solution out of, but PP fibers (3–7 μm) produced by electrospinning from melt showed an oil sorption capacity of 80–129 g/g [16]. PIB is amorphous and can be dissolved in most non-polar organic solvents, therefore it is suitable for centrifugal spinning. Partially crosslinked porous butyl rubber (a copolymer of isobutylene and 2–3% isoprene) sheets showed oil sorption of 8 g/g for olive oil and ~25 g/g for crude oil [22].

Poly(styrene-*b*-isobutylene-*b*-styrene) triblock copolymer (SIBS) is a synthetic thermoplastic elastomer. Some of its remarkable properties are its biostability and fatigue resistance, and it is mostly considered a biomaterial [23]. It became the polymer of choice for a drug-eluting coating on the TAXUS™ coronary stents [24]. Unlike butyl rubber, which in general requires chemical crosslinking for its applications, SIBS is physically crosslinked by nature. SIBS is phase-separated into a rubbery PIB phase (blocks) and a glassy PS phase at room temperature. The PS blocks act as physical crosslinks, granting the material creep resistance and enhanced mechanical properties compared to unvulcanized butyl rubber [23]. Due to the physical crosslinks, SIBS is also recyclable as opposed to butyl rubber. Electrospinning has been used to produce fibers from SIBS. Liu et al. acquired non-circular fibers with a width of 700–1400 nm by electrospinning a 10% *w*/*w* polypyrrole/SIBS blend solution in tetrahydrofuran, with iron(III) p-toluenesulfonate hexahydrate additive for increased solution conductivity. They reported a volumetric flow rate of 1 mL/h [7]. In another study, electrospinning of a 13% *w*/*w* solution of SIBS mixed with carbon nanotubes yielded branched fibers of 50–300 nm in diameter [25]. Lim et al. produced SIBS fibers of 0.3–2 μm (the size increased with concentration, 10–30% *w*/*w,* respectively). The fiber mat had a WCA of 146° [26]. Centrifugal spinning of SIBS at 4500–6300 rpm and a flow rate of 150 mL/h yielded fibers with average diameters of 4–5 μm [27].

The aim of this work was to optimize the centrifugal spinning process to manufacture SIBS fibers. Furthermore, the oil absorption capability of the produced fiber mats was investigated and compared to literature data. 

## 2. Materials and Methods

### 2.1. Materials

Poly(styrene-*b*-isobutylene-*b*-styrene) (SIBS, Sibstar 073T, M_n_ = 65,000 g/mol, 70/30 *w*/*w* PIB/PS, Kaneka Corporation, Osaka, Japan), tetrahydrofuran (THF, reagent grade, VWR), toluene (reagent grade, VWR), and sunflower oil (food grade) were used as received. Tap water was used without any additional treatment.

### 2.2. Solution Preparation

In order to determine the optimal centrifugal spinning conditions, different polymer concentrations and solvent systems were employed. SIBS solutions of 20, 25, and 30% *w*/*w* were prepared by dissolving SIBS pellets in THF at room temperature. Another series of solutions was also prepared from 25% *w*/*w* SIBS and varying THF/toluene mass ratios (90/10, 80/20, 70/30, 60/40, 50/50, 25/75, 0/100). The solutions were prepared using a magnetic stirrer (ARE, Fisher Scientific, Waltham, MA, USA) at 500 rpm at standard laboratory conditions. The dissolved SIBS resulted in a viscous hazy solution after ~1 h of stirring.

### 2.3. Centrifugal Spinning

**Figure 1:**
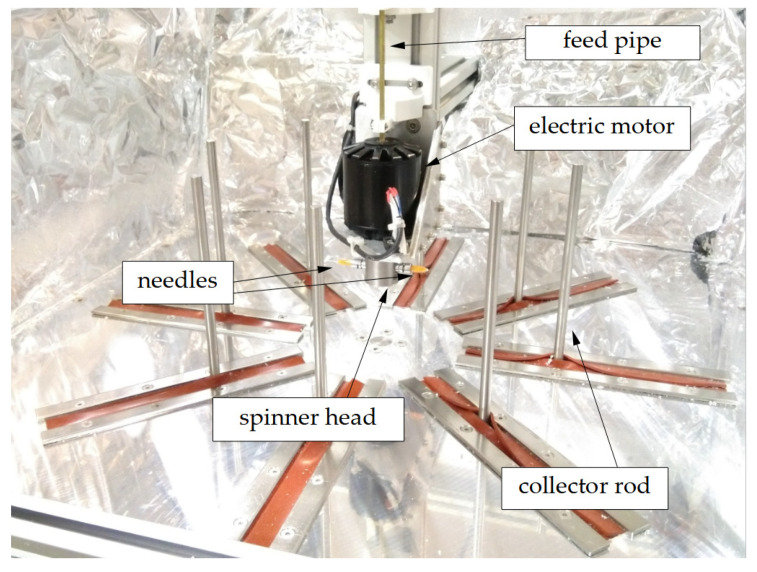
Centrifugal spinning setup.

The centrifugal spinning setup used in the experiments was a custom-made setup (Figure 1) [28]. A small chamber (referred to as spinner head from here on) was connected to an electric motor, which was capable of rotating up to 15,000 rpm. The spinner head had two threaded holes in the radial direction, and needle adapters were inserted into these. The collector consisted of 8 stainless steel rods that could be moved radially direction, effectively modifying its diameter. A copper pipe went through the motor shaft, containing a PTFE tube, through which the polymer solution could be fed into the spinner head.

Centrifugal spinning was carried out in the range of 4000–9000 rpm in 1000 rpm increments, with a 25 G needle (0.25 mm inner diameter) and a 100 mm distance between the collector and the tip of the needle. The solution was fed into the spinner head by a syringe pump (KD Scientific, Holliston, MA, USA) at a controlled volumetric flow rate of 60.5 mL/h. All experiments were carried out at room temperature (~20 °C) and 30–50% relative humidity. The spinning experiments were performed for 1 min. The samples were coded based on the solution concentration, followed by the THF and toluene mass ratios, and the rotational speed times 10^−3^, e.g., 25-T80/t20-8, is the ID of the sample spun from a 25% *w*/*w* solution, where the solvent ratio was 80/20 *w*/*w* THF/toluene, and centrifugal spinning was carried out at 8000 rpm.

### 2.4. Scanning Electron Microscopy (SEM)

SEM imaging was performed with a JEOL JSM-5200 (Tokyo, Japan) scanning electron microscope at 1 kV acceleration voltage on uncoated samples. The SEM images were collected on neat, non-sputter-coated, samples, whereas the fiber sizes, d, were calculated based on 50 measurements on three SEM images taken at different parts of the samples. The open-source ImageJ (version 1.52a, National Institutes of Health, Bethesda, MD, USA) software was used to carry out the measurements. Since the generated fibers do not follow a normal distribution, instead of average size and standard deviation, box plots were used. These provide more accurate information regarding the distribution while also taking outliers into account. To quantify the fiber dimensions, the median values given by the box plots were used.

### 2.5. Viscosity Measurement

The dynamic viscosity of the SIBS solutions was measured with an IKA ROTAVISC lo-vi viscometer (IKA, Königswinter, Germany). The measurements were performed at a shear rate of $\dot{\gamma }$ = 25.5 1/s at 20 °C. The viscosity values were recorded 1 min after turning on the rotation of the spindle.

### 2.6. Contact Angle (CA) Measurement

Droplets of 0.02 mL water or 0.015 mL oil were placed on top of fiber mat samples. Magnified images of the droplets were acquired with a Nikon SMZ645 (Tokyo, Japan) optical microscope. Water contact angle (WCA) values were acquired from the average of five measurements.

### 2.7. Oil Absorption Test

Specimens of 50 ± 3 mg (average thickness of 1 ± 0.1 mm) were cut from the fiber mat and placed in beakers, each containing 40 mL water and 10 mL sunflower oil. The viscosity of the sunflower oil was measured to be 85.3 mPa·s at 21 °C at a shear rate of $\dot{\gamma }$ = 25.5 1/s. The soaking was carried out for 1, 5, 10, 30, and 60 min, repeated 3 times, amounting to a total of 15 samples. After removal, the samples were left to drain for 20 s before weighing. An additional 3 samples were placed in pure water as controls for 60 min and, after that, drained for 20 s.

A second series of experiments were performed in a near-identical fashion. The only difference was that instead of using different specimens for the different soaking times, the same specimens were reused each time. When a target time interval was reached, the specimens were removed from the beakers, weighed, and placed back into their respective beaker for continued soaking.

### 2.8. Relative Density Calculation

The absolute density of a fiber mat specimen was calculated by dividing its mass by its volume. The relative density was acquired with the following formula:

(1) $$\rho_{rel}=\frac{\rho_{abs}}{\rho_{bulk}}$$

where *ρ_bulk_* = 0.954 g/cm^3^, the bulk density of SIBS.

## 3. Results and Discussion

### 3.1. Viscosity Measurement

The measured dynamic viscosity value for the 20% *w*/*w* solution was 50 mPa·s, 124 mPa·s for the 25% solution, and 360 mPa·s for the 30% solution (Table 1). The 25% *w*/*w* solutions containing toluene showed a slight increase in the viscosity: with the increase in toluene concentration, the 90/10 (THF/toluene) was at 140 mPa·s, while the 0/100 (THF/toluene) solution was at 160 mPa·s. The intermediary values were in the 130–150 mPa·s range. Upon repeated measurements, a 10 mPa·s fluctuation was observed within the results for the same solution, so the difference between the solutions containing 100% THF and 100% toluene may be even lower than what the measured values suggest. On the other hand, the increase in viscosity with SIBS concentration was more significant, almost tripling with each step.

### 3.2. Fiber Morphology

The centrifugal spinning of SIBS from its solutions in THF was successful throughout the selected rotational speeds. A summary of the fiber data can be seen in Table 2.

**Figure 2:**
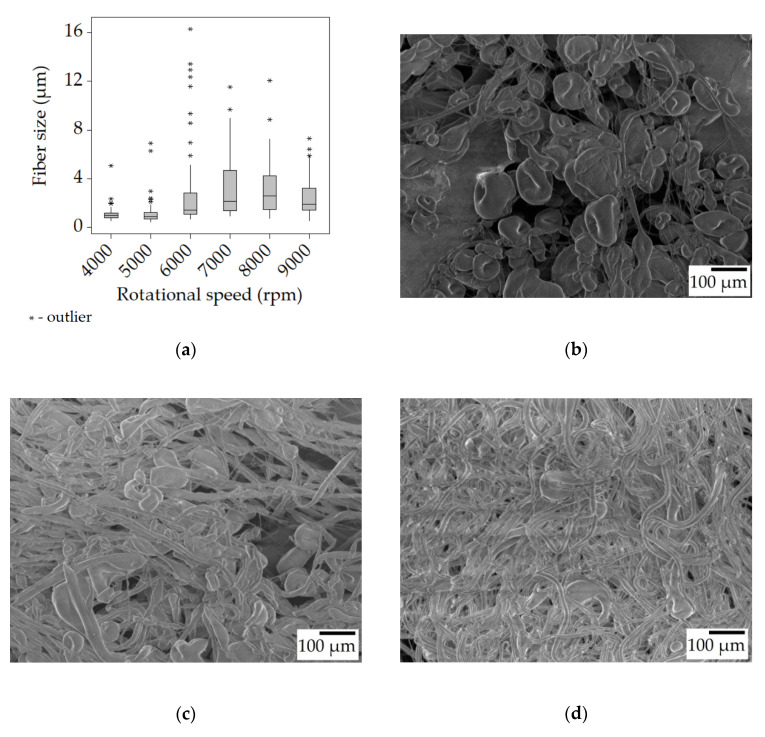
Fiber morphology of the fiber mats spun from the 20% *w*/*w* solution. Box plot of the fiber size data (**a**); SEM micrographs of fibers spun at 4000 rpm (**b**), 6000 rpm (**c**), and 8000 rpm (**d**) at ×100 magnification.

It can be seen that the fibers acquired from the 20% *w*/*w* SIBS solution change in size rather significantly while going from 4000 rpm to 9000 rpm. Figure 2a indicates an increase in size up to 7000 rpm, from 0.95 μm to 2.16 μm, after which there was a slight decrease (not necessarily reflected by the median values). What the box plot does not show is that the fibers produced at 4000–7000 rpm contain a large number of beads, while the 8000–9000 rpm samples have fewer beads. Beading is generally associated with small fiber size. This is due to a force balance issue between surface tension (that facilitates bead formation), tensile forces (that stretch the fibers), and viscoelastic effect (that counteracts both) [29]. When the surface tension is strong compared to the other effects, the majority of the material is concentrated in the beads, leaving the fiber segments between the beads thin.

At 4000 rpm, there was a high bead density (~210 beads/mm^2^, Figure 2b) accompanied by thin and uniform fibers. By increasing the rotational speed, the tensile forces also increased, gradually overcoming the surface tension and leading to a decrease in bead density (Figure 2c,d).

The box plots also show the distribution of the fibers. As the speed increased, the spread also increased along with the median fiber size, as suggested by the longer upper quartiles, while the other 50% of fibers still occupied a relatively narrow range below the median value. Another thing to note is the presence of outliers, represented by the * symbols. In each case, there were fibers whose diameter was significantly larger than the majority. It has been shown that in the initial ~20 s of the centrifugal spinning larger fibers are created, and over time, the fiber size decreases, plateauing at a certain value [30].

**Figure 3:**
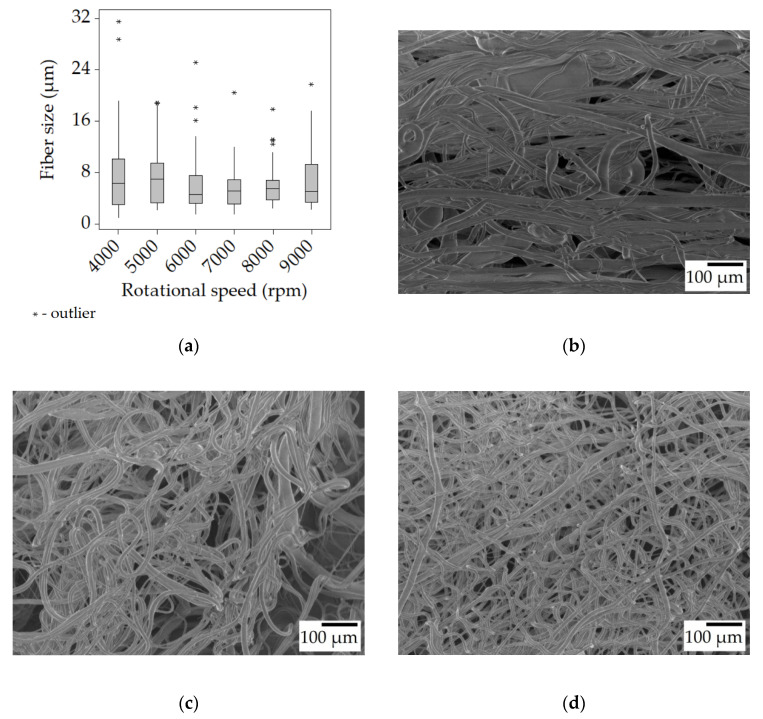
Fiber morphology of the fiber mats spun from the 25% *w*/*w* solution. Box plot of the fiber size data (**a**); SEM micrographs of fibers spun at 4000 rpm (**b**), 6000 rpm (**c**), and 8000 rpm (**d**) at ×100 magnification.

Looking at the samples spun from the 25% *w*/*w* solution, it is clear that the average fiber size increased compared to the 20% *w*/*w* solution. Even the lowest median value of 4.6 μm is almost twice as high as the highest (2.6 μm) in the previous case. The dynamic viscosity of the 25% solution was 124 mPa·s, nearly three times higher than that of the 20% one. This in turn leads to higher viscoelastic forces that hinder the elongation of the fibers during spinning while also preventing bead formation. While the fibers spun at 4000 and 5000 rpm did contain the same beads (Figure 3b), at 6000 rpm and above, no beads were observed (Figure 3c). There was also a trend of decreasing fiber size with increasing rotational speed, the only clear exception being the very last value at 9000 rpm, but this may be due to incorrect sampling. When establishing the trend, not only were the median values considered, but the distribution (the Q4 whiskers becoming shorter) and the outliers too. As the rotational speed increases, so does the centrifugal force acting upon the polymer jet, which is the tensile force in this case, causing the formation of thinner fibers.

**Figure 4:**
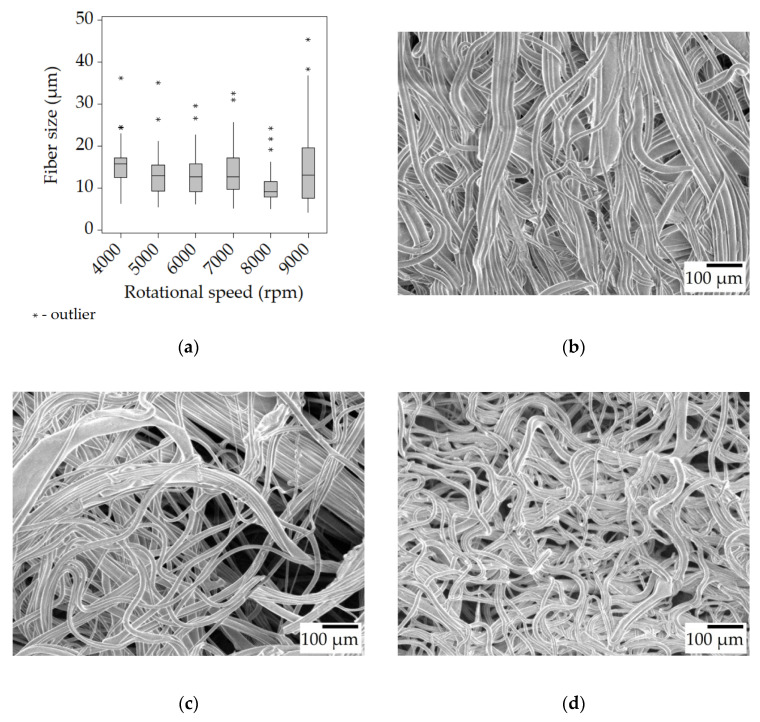
Fiber morphology of the fiber mats spun from the 30% *w*/*w* solution. Box plot of the fiber size data (**a**); SEM micrographs of fibers spun at 4000 rpm (**b**), 6000 rpm (**c**), and 8000 rpm (**d**) at ×100 magnification.

With the 30% *w*/*w* solution, more of the same can be observed but at a higher degree, thanks to its higher viscosity of 350 mPa·s. The median fiber sizes became even larger, d > 10 μm, compared to the results of the 25% *w*/*w* solution. The fiber size decreases with the increase in rotational speed is less pronounced than before, or outright non-existent. The higher viscoelastic force combined with the reduced amount of solvent, and thus quicker drying is the most likely explanation for the large fiber size. A couple of beads formed during spinning at 4000 rpm, but otherwise, the samples contained smooth fibers (Figure 4b–d).

**Figure 5:**
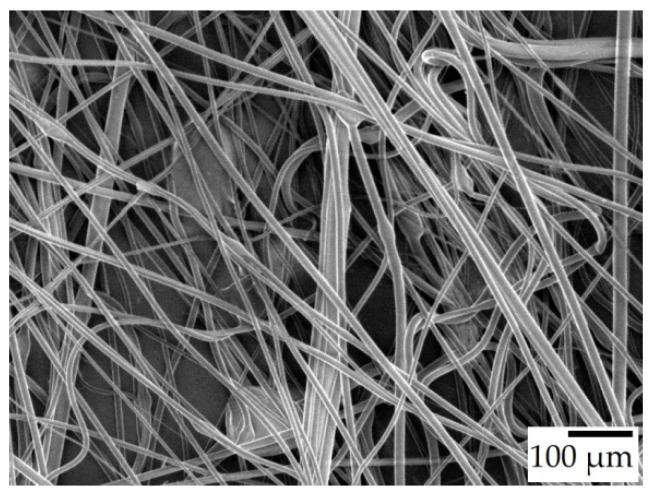
SEM micrograph of 30-T100/t0-6 in their stretched state, ×100 magnification.

Throughout the experiments, it was observed that the generated fibers stretched between the rods of the collector, forming a polygonal shape instead of a circular one. When the fibers were removed from the collector, they shrank in length noticeably, indicating that the fibers were collected in a stretched state on the collector. Some of the fibers were carefully fixed onto a carrier that allowed the fibers to retain their stretched state. SEM imaging on these samples suggested that there was indeed a diameter increase once the fiber mats were taken off the collector. Fibers from the 30% *w*/*w* solution spun at 6000 rpm went from 8.78 to 12.54 μm, and the ones spun at 7000 rpm from 9.33 to 12.56 μm. This means that freshly spun fibers were under tension, and could not relax possibly due to drying out too quickly. Figure 5 shows an SEM image of a sample from 30-T100/t0-6 that was carefully gathered from the collector. The majority of these fibers are completely straight, while after removal from the collector, the fibers became wavy, as seen in Figure 4c.

In order to address the premature drying of the fibers, a part of the highly volatile THF was replaced with toluene. During the preliminary study with the toluene-containing solutions, it was found that a THF/toluene mass ratio of 25/75 and 0/100 yielded only droplets at 8000 rpm. The spinning from a solution of 50/50 solvent mass ratio resulted in beaded fibers. This indicates that the solution of SIBS in toluene has a higher surface tension that the one with THF, as the spinning from the 25% *w*/*w* solution in pure THF produced smooth fibers above 5000 rpm. Thus, a second series of solutions were prepared, with solvent compositions ranging from 90/10 to 60/40 *w*/*w* THF/toluene, and were successfully spun into fibers. The results can be seen in Table 3.

**Figure 6:**
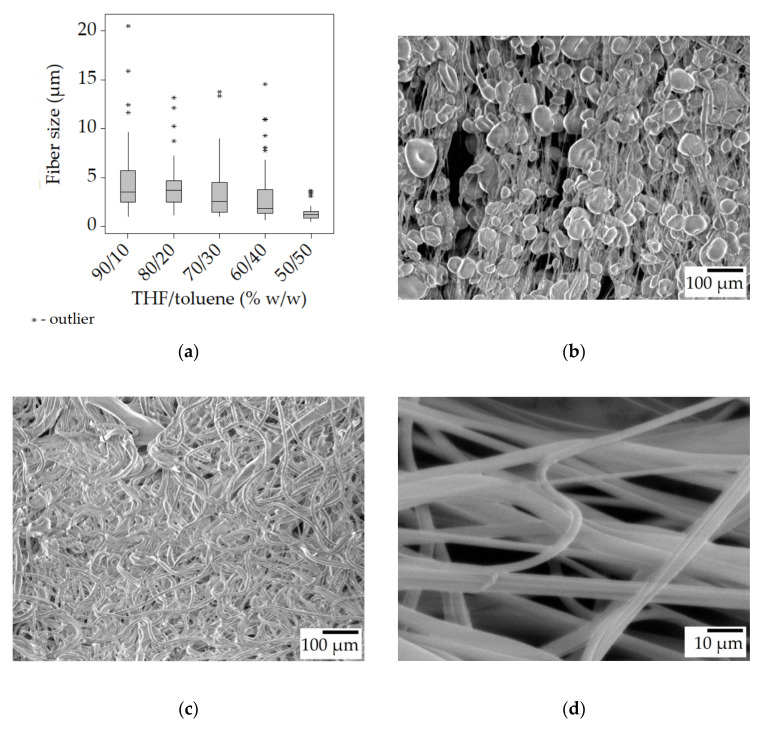
Fiber morphology of the fiber mats spun from the 25% *w*/*w* solution with added toluene at 8000 rpm. Box plot of the fiber size data (**a**); SEM micrographs of 25-T50/t50-8 (**b**), 25-T80/t20-8 (**c**) at ×100 magnification, and the stretched 25-T80/t20-8 at ×1000 magnification (**d**).

The 100/0 (THF/toluene) sample was borrowed from the previous series and is used as a basis for comparison. The data show that as the toluene concentration increased there was a decrease in fiber size from roughly 5 μm to 2 μm. At the 70/30 THF/toluene ratio, beads started to appear. At the 50/50 THF/toluene ratio, beaded fibers formed (Figure 6b), paired with the usual small fiber size and narrow distribution (Figure 6a). Overall, regardless of the solution composition, the smallest bead-free fibers were in the range of 3–4 μm. In contrast, the reported fiber sizes for electrospun SIBS ranged from 300 nm to 2 μm [26].

The smaller fiber sizes (when there was no beading) can be attributed to the longer drying times. Toluene is less volatile than THF, and it is retained longer by the jet, allowing ample time for elongation during spinning, and also for relaxation once on the collector. The measurements seem to support this idea, as the fibers collected in the stretched state had a median size of 0.2–0.3 μm (6–10%) lower than in the unstretched state, while at 60/40 and 50/50 solvent ratios, there was no difference at all. The acquired fibers had a smooth surface (Figure 6d).

**Figure 7:**
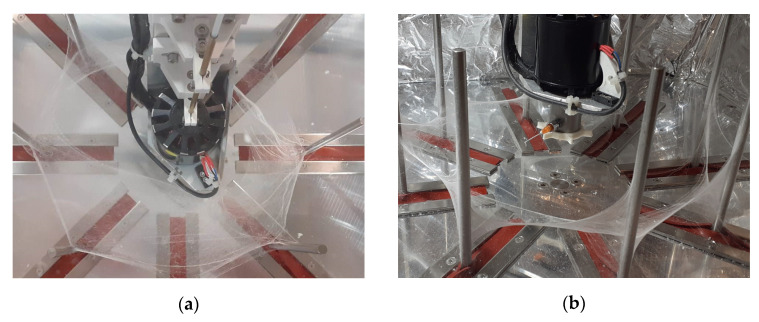
Fibers stretched between the collector and the motor at 100% THF (**a**); fibers gathered on the collector at 50/50 THF/toluene (**b**).

The fibers spun from the solution in THF yielded fibers that barely got attached to the collector, and rather, they were stretched between the collector and the motor holder (Figure 7a). In contrast, when the THF/toluene solvent mixture was used, at higher toluene concentrations (30–50%), the fibers tended to gather on the collector (Figure 7b).

Considering these results, the condition of the 25% *w*/*w* SIBS solution in 80/20 *w*/*w* THF/toluene, and 8000 rpm was chosen to produce a larger quantity of fibers for further experimentation. Under these conditions, smooth fibers were acquired with the smallest size and a relatively narrow size distribution.

### 3.3. Productivity

**Figure 8:**
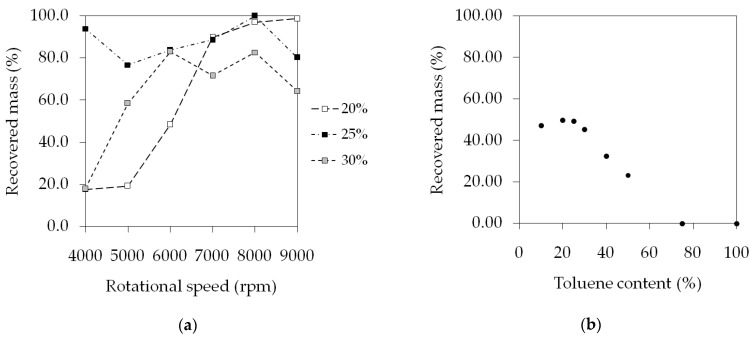
Productivity data of the centrifugal spinning of the THF solutions at various concentrations and speeds (**a**), and the THF/toluene samples at various solvent ratios (**b**).

Centrifugal spinning setups are capable of high productivity, reaching a solution flow rate of 100–300 mL/h (20–70 g fibers/h, depending on the concentration) [12,14]. In our case, the syringe pump was feeding the solution into the spinner head continuously in a controlled manner at a lower, 60.5 mL/h flow rate. Figure 8 shows how much of the SIBS found in the dispensed solutions were processed into fibers. At 20% *w*/*w* and 4000 rpm, the conversion was 17.6%, 1.9 g/h, compared to the theoretical maximum of 10.9 g/h because of the presence of beads and droplets. As the speed increased, so did the amount of recovered material, reaching 100% at 9000 rpm. The 25% *w*/*w* condition behaved somewhat differently. Because of the higher viscosity, the bead/droplet formation was not very pronounced, and thus a higher percentage of the SIBS got stuck on the collector. With the 30% *w*/*w* solution, the conversion peaked at 83%, 13.5 g/h and remained consistently lower than in the previous cases. A possible explanation for this is that the higher viscosity of the solution slowed down the emission, causing accumulation in the spinner head.

In the case of the solutions in the THF/toluene mixture, there seemed to be a reverse correlation between the amount of collected fiber and the toluene concentration (Figure 8b). The explanation, once again, is the increased tendency for droplet formation as the percentage of toluene got higher in the samples. Short strands of fibers were seen floating away in the air during spinning in the case of 50% toluene content and above. It appears that the bead formation caused the break-up of the fibers into short segments. The highest recovery rate of 49.8%, 6.78 g/h out of 13.6 g/h, was observed at the 80/20 THF/toluene ratio.

It should be noted that the run time of these experiments was 1 min, so the small portions of fibers that could not be gathered may have had a significant influence on the results. In order to produce fibers for further use, 9 mL of 25% *w*/*w* SIBS in 80/20 THF/toluene solution was spun at 8000 rpm. The duration of the spinning was 8 min and 40 s, during which 1.8 g dry fibers were acquired, equating to 12.51 g fibers/h. The volumetric flow rate provided by the syringe pump was 60.5 mL/h, as before, and with the 25% *w*/*w* solution that translates to 13.77 g SIBS/h, meaning that the overall recovery percentage was 90.8%.

### 3.4. Contact Angle Measurement

**Figure 9:**
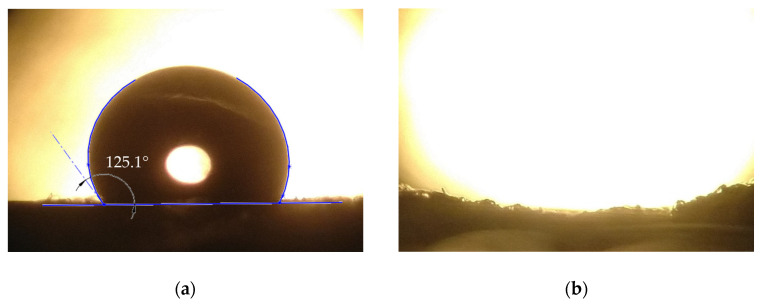
SIBS fiber mat after a water droplet (**a**), and an oil droplet (**b**) being placed on it. The oil droplet cannot be seen, which is due to it being absorbed by the mat.

Water and oil contact angles were measured on the fiber mat produced from the 25% *w*/*w* SIBS solution with 80/20 (THF/toluene) solvent system. An image of a water droplet placed on top of the fiber mat can be seen in Figure 9a. The average WCA was determined to be 129.3 ± 8.1° from 5 measurements.

When it comes to WCA, not only do the properties of the material have an influence, but also any occurrence of micro-, or nanostructures on the surface of the specimen. These phenomena were studied in detail, and the Wenzel and Cassie–Baxter models are used to explain them [31,32]. Previous measurements showed that the WCA on a cast SIBS sheet is ~91° [27]. However, due to the possibility of the formation of air pockets on the surface of the fiber mat, the WCA became much higher than in the case of the bulk material, entering the highly hydrophobic range. There was no oil contact angle to speak of (Figure 7b), as the oil droplet was fully absorbed by the fiber mat within one second. These results demonstrate the hydrophobic and oleophilic nature of the SIBS fiber mat. Reported WCA values for highly absorbent (30–900 g/g) fiber mats are usually above 140° [10,20,33]; however, oil absorption of 30 g/g was also achieved, with fibers mats having an average WCA of 116° [21].

### 3.5. Oil Sorption

**Figure 10:**
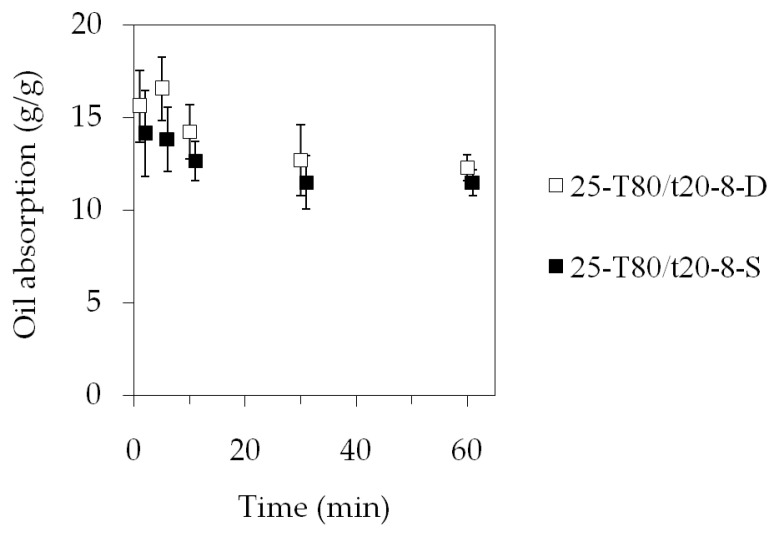
Oil absorption as function of time. The data points in the 25-T80/t20-8-S dataset were shifted by 1 min in order to prevent the overlapping of the error bars.

Five sets of three fiber mat specimens (25-T80/t20-8-D) were placed in beakers containing water and oil. The first set was taken out after 1 min, and the successive ones at 5, 10, 30, and 60 min. The results showed that the fiber mat samples soaked up on average a maximum of 16.5 g oil/g fiber (Figure 10). There seemed to be a spike at the beginning, but after 5 min, the retained oil amount was decreasing, reaching a stable 12.5 g/g. When the specimens were placed on the surface of the oil, they were completely soaked through, sank, and spread out in the oil within 5 s. As time progressed, the samples visibly started to disperse in the oil. By 60 min, the fiber mats started to somewhat lose their structural integrity, and had a swollen appearance right after being lifted out from the oil. This is probably due to the high affinity of the PIB with the oil, and the PIB phase began swelling in the oil, as is typical for rubbers. There were also small pieces of the fiber mat samples floating in the oil that could not be collected, which can lead to a seemingly decreased oil retention.

In order to exclude the possible effect of the difference between the specimens, a second series of measurements was carried out, and this time, the same three specimens were reused each time. The measured dataset is marked with 25-T80/t20-8-S in Figure 10. It can be seen that the general trend remained the same, i.e., the measurements indicated that there was an initial fast uptake of 14 g/g, and over time, the amount of oil absorbed into the fiber mat specimens decreased to 11.5 g/g.

Since actual oil spills involve large quantities of oil, the sorbents should be abundant and cheap. As such, biomass (straw, rice husk, sawdust), and recycled polymer waste (PP, polyurethane) are most commonly used in these situations [34]. Experiments showed that untreated biomass had an absorption capacity of 0.5–7 g crude oil/g sorbent [34,35], while some other natural materials, such as kapok or cotton fibers, could soak up 30–60 g/g [34,36]. Sorption tests with commercial PP fibrous pads showed an uptake of 14 g crude oil/g PP [37]. Woven textiles are generally used for oil–water separation (such as filtration), and not as a sorbent [38]. In the testing of synthetic electrospun/centrifugally spun fibers, crude oil is rarely used; instead, tests are carried out with motor oil, vacuum pump oil, or food-grade oils. Le et al. showed that electrospun PVC fibers absorbed twice as much motor oil than crude oil [39]. It was also found that the absorption capacity for food-grade oils is 20% lower for PS [33], and 30–40% lower for PP fibers [16], compared to motor oil. A couple of examples of the performance of non-woven fiber mats in the absorption of food-grade oils are as follows: 25 g/g sunflower and vegetable oil, 30 g/g peanut oil in the case of porous PLA fibers [21], 30 g/g peanut oil for rough PS (3.7 μm size) fibers, 110 g/g for porous PS (0.5 μm size) [33], 30–45 g/g vegetable oil for porous PS fibers [20], and 100–500 g/g sunflower oil with porous PS fibers [10]. Table 4 summarizes the above-mentioned results. It has to be noted that SIBS has a higher price than PS, PLA, PP, or PVC, especially if the later materials are recycled. SIBS is a thermoplastic elastomer, and the others, excluding PLA, are mass-produced thermoplastics. However, due to the fast absorption kinetics and excellent chemical resistance of SIBS, it could be used in some special applications where fast absorption is essential. 

**Figure 11:**
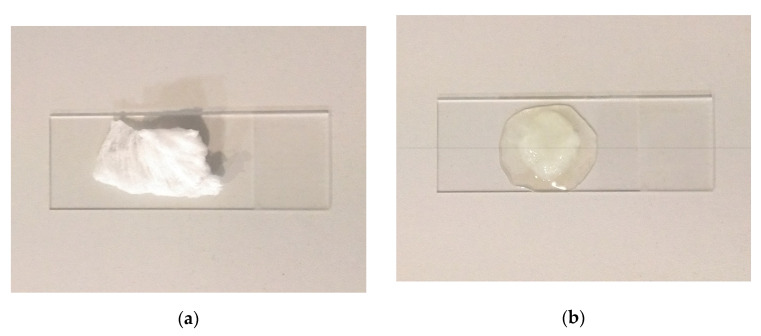
SIBS fiber mat specimen before (**a**) and after (**b**) oil sorption.

Figure 11 shows a specimen before being placed in the oil and after 5 min of sorption. The specimen in Figure 11b had been sitting on the glass slide for approximately 10 min when the picture was taken. The oil spot around the specimen indicates that over time some of the oil seeps out of the fiber mat, and thus it cannot retain all the absorbed oil.

The relative density of the fiber mat was calculated to be ρ_rel_ = 0.092 g/cm^3^, meaning that a certain volume of fiber mat weighed ~9% of what an identical volume of bulk SIBS would have (or that 91% of the volume was air). At this relative density, the average volume of a 50 mg sample used in the absorption study would be 0.57 cm^3^. At an average of 16.5 g/g oil absorption, the amount of oil taken up by 50 mg fiber is 0.825 g or 0.9 mL. Combining these calculations, we find that the oil uptake of the SIBS mats was 1.55 cm^3^ oil/cm^3^ fiber mat. Porous fibers have been shown to better absorb oil when compared to smooth fibers [10]. The presence of pores offers a higher surface area to the fiber mat, thus increasing the adhesion between the polymer and the oil, which can be crucial for less oleophilic materials. SIBS has a high affinity to oil (as indicated by the lack of an oil contact angle), so the main advantage of the porous fibers would be the decreased relative density of the mat (assuming the same fiber diameter).

Published data show that in general, over the course of 1–2 h, the absorbed oil amount increases until it reaches a plateau, while the initial uptake is 70–80% in 5 min [39], 45–70% in 1 min [33], or 25–90% in 1 min [10]. In contrast to these findings, the SIBS fibers achieved near-maximum uptake within the first minute. Butyl rubber sorbents and PP fibers (both of which are very similar to PIB) also showed a near-instantaneous saturation, reaching their sorption limit in 5–60 s, depending on the oil type [22].

The samples placed in pure water picked up a minuscule amount of mass in the form of well-defined droplets clinging to the surface of the fiber mat. The overall water uptake of the samples was 0.04 g/g.

## 4. Conclusions

Centrifugal spinning is a strong competitor in the field of fiber production, as it is able to produce polymeric fibers at an increased production rate and from non-conductive polymer solutions. Smooth SIBS fibers were successfully produced with centrifugal spinning at various conditions. Optimal conditions were achieved with the 25% *w*/*w* SIBS solution concentration using a THF/toluene solvent system at an 80/20 *w*/*w* ratio and 8000 rpm rotation speed. The median fiber size under these conditions was 3.68 μm. Contact angle measurements showed that the fiber mats are highly hydrophobic with a WCA of 129° while being completely wetted by sunflower oil. The maximum observed oil uptake was 16.5 g/g after 5 min, decreasing to 12.5 g/g by 30 min. The performance of these solid fibers was comparable with PLA and PS solid fibers for food-grade oil sorption, published in the literature, but much lower than the fiber mats made out of porous fibers. On the other hand, the SIBS fibers demonstrated unusually quick sorption characteristics, reaching 94–100% already at 1 min. The above-mentioned data indicate that PIB-based thermoplastic elastomers are promising candidates for oil separation applications due to their fast sorption capabilities.

Further work is necessary to investigate how to enhance the oil sorption capacity of the SIBS fiber mats, and find out what would be the ideal situation for its applications. A possible way to improve upon the concept is the creation of porous fibers, which would increase the specific volume of the fiber mats, and thus their capacity to capture more oil g/g. 

## Figures and Tables

**Table 1:** Dynamic viscosity values of the SIBS solutions.

SIBS Concentration (% *w*/*w*)	THF/tol (*w*/*w*)	Dynamic Viscosity (mPa·s)
20	100/0	50.1
25	100/0	124.3
	90/10	139.3
	80/20	135.4
	70/30	151.7
	60/40	144.6
	50/50	157.7
	25/75	129.8
	0/100	161.1
30	100/0	360.4

**Table 2:** Median fiber diameter of SIBS fibers spun from solutions in THF in function of solution concentration and rotational speed.

Sample ID	SIBS Concentration (% *w*/*w*)	Rotational Speed (rpm)	Fiber Size (μm)	Morphological Features
20-T100/t0-4	20	4000	0.95	beads
20-T100/t0-5		5000	0.86	beads
20-T100/t0-6		6000	1.43	beads
20-T100/t0-7		7000	2.16	beads
20-T100/t0-8		8000	2.60	few beads
20-T100/t0-9		9000	1.89	few beads
25-T100/t0-4	25	4000	6.37	few beads
25-T100/t0-5		5000	6.98	few beads
25-T100/t0-6		6000	4.60	bead-free
25-T100/t0-7		7000	5.16	bead-free
25-T100/t0-8		8000	5.48	bead-free
25-T100/t0-9		9000	5.09	bead-free
30-T100/t0-4	30	4000	15.66	few beads
30-T100/t0-5		5000	12.83	bead-free
30-T100/t0-6		6000	12.54	bead-free
30-T100/t0-7		7000	12.56	bead-free
30-T100/t0-8		8000	9.11	bead-free
30-T100/t0-9		9000	13.02	bead-free

**Table 3:** Median fiber diameter of SIBS fibers spun from solutions in THF/toluene at 8000 rpm.

Sample ID	Concentration (% *w*/*w*)	THF/tol (*w*/*w*)	Fiber Size (μm)	Morphological Features
25-T100/t0-8	25	100/0	5.48	bead-free
25-T90/t10-8		90/10	3.52	bead-free
25-T80/t20-8		80/20	3.68	bead-free
25-T70/t30-8		70/30	2.50	few beads
25-T60/t40-8		60/40	1.83	few beads
25-T50/t50-8		50/50	1.17	beads

**Table 4:** Oil sorption experiment results from literature.

Material	Method	Used Oil	Sorption Capacity (g/g)	Ref.
PVC	Electrospinning	Motor oil	~40	[39]
PP	Needleless melt electrospinning	Peanut oil	~140	[16]
PLA	Centrifugal spinning	Sunflower oil/vegetable oil	25	[21]
		Peanut oil	30	
PS	Electrospinning	Peanut oil	30	[33]
Porous PS		Peanut oil	110	
Porous PS	Centrifugal spinning	Vegetable oil	30–45	[20]
Porous PS	Electrospinning	Sunflower oil	100–500	[10]

## Data Availability

The data presented in this study are available on request from the corresponding author.

## References

[1] Asif Z., Chen Z., An C., Dong J. (2022). Environmental Impacts and Challenges Associated with Oil Spills on Shorelines. JMSE.

[2] Beyer J., Trannum H.C., Bakke T., Hodson P.V., Collier T.K. (2016). Environmental Effects of the Deepwater Horizon Oil Spill: A Review. Mar. Pollut. Bull..

[3] Ventikos N.P., Vergetis E., Psaraftis H.N., Triantafyllou G. (2004). A High-Level Synthesis of Oil Spill Response Equipment and Countermeasures. J. Hazard. Mater..

[4] Ivshina I.B., Kuyukina M.S., Krivoruchko A.V., Elkin A.A., Makarov S.O., Cunningham C.J., Peshkur T.A., Atlas R.M., Philp J.C. (2015). Oil Spill Problems and Sustainable Response Strategies through New Technologies. Environ. Sci. Process. Impacts.

[5] Ge J., Zhao H.-Y., Zhu H.-W., Huang J., Shi L.-A., Yu S.-H. (2016). Advanced Sorbents for Oil-Spill Cleanup: Recent Advances and Future Perspectives. Adv. Mater..

[6] Reneker D.H., Yarin A.L. (2008). Electrospinning Jets and Polymer Nanofibers. Polymer.

[7] Liu X., Chen J., Gilmore K.J., Higgins M.J., Liu Y., Wallace G.G. (2010). Guidance of Neurite Outgrowth on Aligned Electrospun Polypyrrole/Poly(Styrene-β-Isobutylene-β-Styrene) Fiber Platforms. J. Biomed. Mater. Res. Part A.

[8] Jindal A., Puskas J.E., McClain A., Nedic K., Luebbers M.T., Baker J.R., dos Santos B.P., Camassola M., Jennings W., Einsporn R.L. (2018). Encapsulation and Release of Zafirlukast from Electrospun Polyisobutylene-Based Thermoplastic Elastomeric Fiber Mat. Eur. Polym. J..

[9] Yan G., Niu H., Lin T. (2019). Needle-Less Electrospinning. Electrospinning: Nanofabrication and Applications.

[10] Chen P.-Y., Tung S.-H. (2017). One-Step Electrospinning to Produce Nonsolvent-Induced Macroporous Fibers with Ultrahigh Oil Adsorption Capability. Macromolecules.

[11] Marjuban S.M.H., Rahman M., Duza S.S., Ahmed M.B., Patel D.K., Rahman M.S., Lozano K. (2023). Recent Advances in Centrifugal Spinning and Their Applications in Tissue Engineering. Polymers.

[12] Vo P.P., Doan H.N., Kinashi K., Sakai W., Tsutsumi N., Huynh D.P. (2018). Centrifugally Spun Recycled PET: Processing and Characterization. Polymers.

[13] Merchiers J., Meurs W., Deferme W., Peeters R., Buntinx M., Reddy N.K. (2020). Influence of Polymer Concentration and Nozzle Material on Centrifugal Fiber Spinning. Polymers.

[14] Skrivanek J., Holec P., Batka O., Bilek M., Pokorny P. (2022). Optimization of the Spinneret Rotation Speed and Airflow Parameters for the Nozzleless Forcespinning of a Polymer Solution. Polymers.

[15] Brown T.D., Dalton P.D., Hutmacher D.W. (2016). Melt Electrospinning Today: An Opportune Time for an Emerging Polymer Process. Prog. Polym. Sci..

[16] Li H., Wu W., Bubakir M.M., Chen H., Zhong X., Liu Z., Ding Y., Yang W. (2014). Polypropylene Fibers Fabricated via a Needleless Melt-Electrospinning Device for Marine Oil-Spill Cleanup. J. Appl. Polym. Sci..

[17] Guo M., Liang H., Luo Z., Chen Q., Wei W. (2016). Study on Melt-Blown Processing, Web Structure of Polypropylene Nonwovens and Its BTX Adsorption. Fibers Polym..

[18] Qi B., Hu X., Cui S., Liu H., Li Y., Li Y., Lu J., Bao M. (2023). Rapid Fabrication of Superhydrophobic Magnetic Melt-Blown Fiber Felt for Oil Spill Recovery and Efficient Oil–Water Separation. Sep. Purif. Technol..

[19] Ellison C.J., Phatak A., Giles D.W., Macosko C.W., Bates F.S. (2007). Melt Blown Nanofibers: Fiber Diameter Distributions and Onset of Fiber Breakup. Polymer.

[20] Doan H.N., Nguyen D.K., Vo P.P., Hayashi K., Kinashi K., Sakai W., Tsutsumi N., Huynh D.P. (2019). Facile and Scalable Fabrication of Porous Polystyrene Fibers for Oil Removal by Centrifugal Spinning. ACS Omega.

[21] Zhang L., Narita C., Himeda Y., Honma H., Yamada K. (2022). Development of Highly Oil-Absorbent Polylactic-Acid Microfibers with a Nanoporous Structure via Simple One-Step Centrifugal Spinning. Sep. Purif. Technol..

[22] Ceylan D., Dogu S., Karacik B., Yakan S.D., Okay O.S., Okay O. (2009). Evaluation of Butyl Rubber as Sorbent Material for the Removal of Oil and Polycyclic Aromatic Hydrocarbons from Seawater. Environ. Sci. Technol..

[23] Pinchuk L., Wilson G.J., Barry J.J., Schoephoerster R.T., Parel J.-M., Kennedy J.P. (2008). Medical Applications of Poly(Styrene-Block-Isobutylene-Block-Styrene) (“SIBS”). Biomaterials.

[24] Kamath K.R., Barry J.J., Miller K.M. (2006). The Taxus^TM^ Drug-Eluting Stent: A New Paradigm in Controlled Drug Delivery. Adv. Drug Deliv. Rev..

[25] Liu Y., Gilmore K.J., Chen J., Misoska V., Wallace G.G. (2007). Bio-Nanowebs Based on Poly(Styrene-β-Isobutylene-β-Styrene) (SIBS) Containing Single-Wall Carbon Nanotubes. Chem. Mater..

[26] Lim G.T., Puskas J.E., Reneker D.H., Jákli A., Horton W.E. (2011). Highly Hydrophobic Electrospun Fiber Mats from Polyisobutylene-Based Thermoplastic Elastomers. Biomacromolecules.

[27] Kantor J., Gergely A.L., Farmos R.L., Hodgyai N. (2022). Poly(Styrene-b-Isobutylene-b-Styrene) Triblock Copolymer Fiber Generation with Centrifugal Spinning, and Its Potential Application in Oil Collection. Proceedings of the 2022 IEEE 22nd International Symposium on Computational Intelligence and Informatics and 8th IEEE International Conference on Recent Achievements in Mechatronics, Automation, Computer Science and Robotics (CINTI-MACRo).

[28] Fábián H., Gergely A. (2022). Design of a High Performance Fiber-Producing Machine. Acta Mater. Transylvanica.

[29] Fong H., Chun I., Reneker D.H. (1999). Beaded Nanofibers Formed during Electrospinning. Polymer.

[30] McEachin Z., Lozano K. (2012). Production and Characterization of Polycaprolactone Nanofibers via Forcespinning^TM^ Technology. J. Appl. Polym. Sci..

[31] Wenzel R.N. (1936). RESISTANCE OF SOLID SURFACES TO WETTING BY WATER. Ind. Eng. Chem..

[32] Cassie A.B.D., Baxter S. (1944). Wettability of Porous Surfaces. Trans. Faraday Soc..

[33] Wu J., Wang N., Wang L., Dong H., Zhao Y., Jiang L. (2012). Electrospun Porous Structure Fibrous Film with High Oil Adsorption Capacity. ACS Appl. Mater. Interfaces.

[34] Al-Majed A.A., Adebayo A.R., Hossain M.E. (2012). A Sustainable Approach to Controlling Oil Spills. J. Environ. Manag..

[35] Pintor A.M.A., Vilar V.J.P., Botelho C.M.S., Boaventura R.A.R. (2016). Oil and Grease Removal from Wastewaters: Sorption Treatment as an Alternative to State-of-the-Art Technologies. A Critical Review. Chem. Eng. J..

[36] Choi H. (1996). Needlepunched Cotton Nonwovens and Other Natural Fibers as Oil Cleanup Sorbents. J. Environ. Sci. Health. Part A Environ. Sci. Eng. Toxicol..

[37] Wei Q.F., Mather R.R., Fotheringham A.F., Yang R.D. (2003). Evaluation of Nonwoven Polypropylene Oil Sorbents in Marine Oil-Spill Recovery. Mar. Pollut. Bull..

[38] Zaarour B., Liu W. (2023). Recent Advances of Textile Sorbents for Oil Spills Cleanup: A Review. J. Ind. Text..

[39] Le Q.P., Olekhnovich R.O., Uspenskaya M.V., Vu T.H.N. (2021). Study on Polyvinyl Chloride Nanofibers Ability for Oil Spill Elimination. Iran. Polym. J..

